# Haploinsufficiency of the Attention-Deficit/Hyperactivity Disorder Risk Gene *St3gal3* in Mice Causes Alterations in Cognition and Expression of Genes Involved in Myelination and Sialylation

**DOI:** 10.3389/fgene.2021.688488

**Published:** 2021-09-28

**Authors:** Olga Rivero, Judit Alhama-Riba, Hsing-Ping Ku, Matthias Fischer, Gabriela Ortega, Péter Álmos, David Diouf, Daniel van den Hove, Klaus-Peter Lesch

**Affiliations:** ^1^Division of Molecular Psychiatry, Center of Mental Health, University of Würzburg, Würzburg, Germany; ^2^Biomedical Network Research Centre on Mental Health (CIBERSAM), Valencia, Spain; ^3^Department of Psychiatry, Psychosomatics and Psychotherapy, University of Würzburg, Würzburg, Germany; ^4^Department of Psychiatry and Neuropsychology, School for Mental Health and Neuroscience, Maastricht University, Maastricht, Netherlands; ^5^Laboratory of Psychiatric Neurobiology, Institute of Molecular Medicine, I.M Sechenov First Moscow State Medical University, Moscow, Russia

**Keywords:** *St3gal3*, sialyltransferase, sialic acid, psychiatric disorders, attention-deficit/hyperactivity disorder (ADHD), prefrontal cortex, hippocampus, mouse model

## Abstract

Genome wide association meta-analysis identified *ST3GAL3*, a gene encoding the beta-galactosidase-alpha-2,3-sialyltransferase-III, as a risk gene for attention-deficit/hyperactivity disorder (ADHD). Although loss-of-function mutations in *ST3GAL3* are implicated in non-syndromic autosomal recessive intellectual disability (NSARID) and West syndrome, the impact of *ST3GAL3* haploinsufficiency on brain function and the pathophysiology of neurodevelopmental disorders (NDDs), such as ADHD, is unknown. Since *St3gal3* null mutant mice display severe developmental delay and neurological deficits, we investigated the effects of partial inactivation of *St3gal3* in heterozygous (HET) knockout (*St3gal3*^±^) mice on behavior as well as expression of markers linked to myelination processes and sialylation pathways. Our results reveal that male *St3gal3* HET mice display cognitive deficits, while female HET animals show increased activity, as well as increased cognitive control, compared to their wildtype littermates. In addition, we observed subtle alterations in the expression of several markers implicated in oligodendrogenesis, myelin formation, and protein sialylation as well as cell adhesion/synaptic target glycoproteins of ST3GAL3 in a brain region- and/or sex-specific manner. Taken together, our findings indicate that haploinsufficiency of ST3GAL3 results in a sex-dependent alteration of cognition, behavior and markers of brain plasticity.

## Introduction

Neurodevelopmental disorders (NDDs) constitute frequent causes of disability among children and adolescents world-wide. Being the most common NDD, attention-deficit/hyperactivity disorder (ADHD, MIM #143465), is defined as a clinically heterogeneous syndrome comprising developmentally inappropriate inattention, hyperactivity, increased impulsivity, and motivational/emotional dysregulation. The syndromal dimensions of inattention and impulsivity are increasingly being recognized as highly persistent into adulthood and are associated with a considerable risk for failure in psychosocial adaptation as well as psychiatric co-morbidity, such as depression and substance use disorders (Bush, 2010; Faraone and Larsson, 2019).

Despite evidence for high heritability, the multifactorial and polygenic nature of ADHD has rendered it challenging to unravel the genetic contribution to each syndromal dimension. Recently, a collaborative effort which assessed a sample of 20,183 individuals with ADHD and 35,191 controls identified ADHD risk variants surpassing genome-wide significance in 12 independent loci (Demontis et al., 2019). This meta-analysis shed light on a locus located on chromosome 1, whose most significant single nucleotide polymorphism (SNP), rs11420276 (*p* = 2.14 × 10^–13^), is located in *ST3GAL3*, a gene encoding the Golgi membrane enzyme beta-galactosidase-alpha-2,3-sialyltransferase-III (ST3GAL3). Of note, a recent genome-wide methylome screening by Walton et al. (2017) also identified *ST3GAL3* as differentially methylated during the course of ADHD. It was pointed out that gene-by-environment (GxE) interaction may moderate *ST3GAL3* expression, impacting epigenetic programming of brain development and maturation. Remarkably, *ST3GAL3* was implicated in general cognitive function in a GWAS meta-analysis (Davies et al., 2018). Together, these findings identify *ST3GAL3* as a putative risk gene for ADHD, specifically for associated impairment of cognitive performance.

In support of this notion, loss-of-function mutations in *ST3GAL3* were previously reported to result in severe non-syndromic autosomal recessive intellectual disability (NSARID), a familial disorder characterized by low cognitive abilities associated with difficulty in learning basic language and motor skills, as well as West syndrome (WS), a severe infantile seizure disorder associated with developmental regression and intellectual disability that can occur as an autosomal recessive familial disorder (Hu et al., 2011; Edvardson et al., 2013; Schnaar et al., 2014; Indellicato et al., 2020), compellingly suggesting that ST3GAL3 function is critical for normal development and functioning of the brain.

ST3GAL3 is mainly involved in the formation of the sialyl epitope on glycoproteins, a process with critical impact on brain function which also determines the functional specificity of glycans (Schnaar et al., 2014). Sialic acids (Sia) are 9-carbon backbone sugars with chemical properties that are well suited for molecular recognition and communication (Yoo et al., 2015). Brain sialoglycans are dominated by gangliosides, sialic acid-bearing glycosphingolipids that constitute approximately 75% of the brain’s sialic acid, whereas sialoglycoproteins constitute the remaining 25% (Yoo et al., 2015).

Sialoglycans are biosynthesized by sialyltransferases (STs), which catalyze the transfer of Sia from its activated nucleotide sugar donor cytidine monophosphate (CMP-Sia) to the terminus of an oligosaccharide chain of a glycoprotein or glycolipid (Wang, 2009; Schnaar et al., 2014). Based on their acceptor substrates, the type of glycosidic linkage they generate (α2-3, α2-6, and α2-8) and their primary saccharide receptor [galactose (Gal), N-Acetyl-D-galactosamine (GalNAc) or Sia] (Wang, 2009; Schnaar et al., 2014), 20 mammalian STs have been described and categorized into four families: β-galactoside α2,3-sialyltransferases (ST3Gal), β-galactoside α2,6-sialyltransferases (ST6Gal), alpha-N-acetylgalactosaminide α2,6-sialyltransferases (ST6GalNAc), and α2,8-sialyltransferases or polysialyltransferases (ST8Sia). Particularly, ST3Gal3 utilizes both Galß1,3GlcNAc and Galß1,4GlcNAc structures (with preference for Galß1,3GlcNAc structure), mainly on glycoproteins as acceptor substrates (Yoo et al., 2015). Additionally, polysialyltransferases (in vertebrates ST8Sia2 and ST8Sia4) attach polysialic acid (PSA) to complex glycans with the first sialic acid.

Among several other proteins, PSA is added to the neural cell adhesion molecule-1 (NCAM1). Attachment of PSA to NCAM1 influences its properties, altering cell motility, neurogenesis, axon outgrowth as well as structural and synaptic plasticity (Galuska et al., 2008; Wang, 2009; Nacher et al., 2013; Schnaar et al., 2014). PSA is also added to other molecules, such as synaptic cell adhesion molecule-1 (SynCAM1, CADM1), Neuropilin-2 or voltage-gated ion channels (Ednie and Bennett, 2012; Mühlenhoff et al., 2013), in specific cell types. The effects of sialylation on these glycoproteins are yet unknown.

Although ST3GAL3 is well characterized in the context of cancer or lung inflammation, little is known about its function in the brain. Yoo et al. (2015) assessed neuroanatomy, brain histology, biochemistry as well as behavior in *St3gal2* and *St3gal3* single and double-null mutant mice. Notably, the authors demonstrated that sialylation of sialoglycoproteins is decreased approximately 50% in *St3gal3*^–/–^ mice. Moreover, the authors reported alterations in different markers linked to oligodendrogenesis and the process of myelination (MBP, Olig2, and MAG). Additionally, they reported reduced motor coordination, marked cognitive deficits and hyperactive behavior in *St3gal3* single-null mice.

The notion that *ST3GAL3* inactivation causes severe intellectual disability with distinguishable developmental, neurological and neuropsychiatric phenotypes led us to predict that more subtle allelic variation of *ST3GAL3* expression may increase the vulnerability to ADHD and related conditions, or predispose individuals to the effects of other genetic and/or environmental risk factors. Since *St3gal3* null mutant mice display severe developmental delay, neurological deficits, including spontaneous seizures, and overall reduced viability, thus limiting behavioral and neurobiological assessment, we here investigated the effects of partial inactivation of *St3gal3* in male and female heterozygous knockout (*St3gal3*^±^) mice with focus on behavior as well as expression of markers linked to sialylation and myelination pathways.

## Materials and Methods

### Generation of *St3gal3*-Deficient Mice

Experiments were performed using a constitutive *St3gal3*-deficient mouse line (B6.129-*St3gal3^TM 1Jxm^*/J) that had been previously generated by Cre-loxP gene targeting to produce the deletion of exon 2 that encodes the ST3GAL3 transmembrane domain (Ellies et al., 2002). Heterozygous *St3gal3*-deficient (*St3gal3*^±^, in the following referred to as HET) were obtained from the Jackson Laboratory (JAX, Bar Harbor, ME, United States) and re-derived by *in vitro* fertilization and implantation in C57BL/6J dams at the Center for Experimental Molecular Medicine (ZEMM), University of Würzburg. After the rederivation, the mouse line was maintained by breeding HET males with *St3gal3*^+/+^ wildtype (WT) females. The genotyping of the *St3gal3* locus was done by polymerase chain reaction (PCR) as described on the Jackson Lab website.^1^ In order to confirm the deletion and deficiency of ST3GAL3, we measured mRNA and protein levels in the mouse brain by quantitative real time PCR (qRT-PCR), as described in 2.5.

### Animal Husbandry

All mice used for this study were housed in standard Makrolon cages at the animal facility of the ZEMM (University Hospital of Würzburg), under a 12 h light/dark cycle with food and water *ad libitum* unless specified otherwise. After weaning, male mice were kept in type IV Makrolon cages (rat type) in groups of 9–10 animals of mixed genotypes, in order to follow the group distribution needed for the behavioral experiments in the Intellicage System (see section “Behavioral Assessment”). This was done to avoid future aggressive reactions between adult male animals coming from different litters due to the territorial behavior that naturally occurs in male mice (Koolhaas et al., 2013), which can be avoided if animals from different litters are mixed at an early age. In the case of female mice, they were kept in type III Makrolon cages in genotype-mixed groups of 3–5 per cage until the start of the behavioral experiments. Animals used for the molecular experiments were housed following the same strategy described above. Experimental procedures of the IntelliCage System were approved by the local authorities of the Government of Lower Franconia and performed in accordance with the guidelines for animal care and use provided by the European Parliament and Council Directive (2010/63/EU).

### Behavioral Assessment

Behavioral tests were performed to assess activity, memory and learning abilities as well as cognitive flexibility and impulsivity in two separate cohorts of adult male (*n* = 18; 9 WT and 9 HET) and female (*n* = 18; 8 WT and 10 HET) mice. Due to the impossibility to test females and males simultaneously, both sexes had to be tested at different time points: the male cohort was 3–4 months of age at the beginning of the testing; the female cohort was 5–7 months old. However, we consider that the age difference between males and females, which is around 2–3 months, is not as high as being considered a main confounding factor (Benice et al., 2006). In addition, all the animals used in the behavioral experiment are full adults that have not started with any aging process which may have underlid strong differences in their performance in the Intellicage (Yanai and Endo, 2021).

**FIGURE 1:**
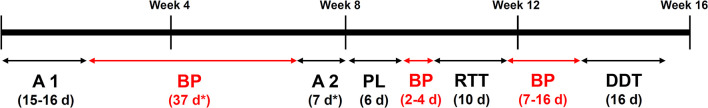
Timeline of the different behavioral experiments performed in this study by the male and female cohort. A, adaptation phase; BP, break phase; PL, place learning; RTT, reaction time task; DDT, delayed discounting task; d, days. *Males only.

Behavioral assessment was carried out using the IntelliCage System (New Behavior, Zurich, Switzerland), an automated home cage system (Krackow et al., 2010; Kobayashi et al., 2013; Fischer et al., 2017). For that purpose, mice were anesthetized with isoflurane and radio frequency identification transponders (Planet ID, Essen, Germany) were implanted subcutaneously in the dorso-cervical region. Animals were returned to their home cages and stayed in observation for recovery for a few days. Afterward, mice were transferred to the Intellicage System, where the following phases/behavioral tasks took place (Figure 1).

#### Adaptation Phase

The adaptation phase allowed us to investigate exploratory behavior in a novel environment. During the first 7 days (spontaneous activity subphase, SA), all IntelliCage doors were open, providing free access to all eight water bottles (two in each corner). Animals that were unable to drink from the bottles 48 h after being introduced in the Intellicage were excluded from the experiment. Afterward, the nosepoke (np) phase took place; during np phase, the mice had to make a np to be able to drink from the water bottles. Likewise, those animals that were unable to learn the task and drink from the bottles after 48 h were excluded from the experiment. In this module, the total number of visits/h, visits without np or licks/h, visits with np/h, visits with licks/h, median number of np and licks per visit, median duration of nosepokes as well as median duration of licking were measured.

Due to technical reasons (forced experimental discontinuity in the animal facility), male mice had to be removed from the Intellicage after the Adaptation Phase and returned to normal cages. Once again in the Intellicage and before the start of the place learning phase, they were subjected to a second Adaptation period, which consisted only in np phase (Figure 1). Results concerning this second Adaptation period are shown in Supplementary Figure 1.

#### Place-Learning Phase

This phase allowed us to evaluate learning and memory abilities as well as cognitive flexibility. In this phase, water was only available for each mouse in one specific corner (termed as “correct”), while the rest of the corners (termed as “incorrect”) did not open the doors even if the mouse had made a nosepoke. Correct corners were assigned depending on the corner preferences of each mouse during the adaptation phase; the least visited corner was assigned as the correct one. To avoid learning by imitation, balance between corner preferences was also taken into account. The first place learning module lasted for three days. This was followed by 3 days of place reversal, in which the rewarded corner was swapped to the opposite one, allowing us to evaluate cognitive flexibility. In this module, correct and incorrect responses were measured and the proportion of correct responses was calculated. The learning criterion was set at 35% of correct responses, that is, random expectation plus 10%.

#### Reaction Time-Task (RTT)

The reaction time-task (RTT) evaluates motor impulsivity and was divided into training and testing phases, which lasted 3 and 5 days, respectively. During the training part, the first np on each visit was followed by a delay period of variable (random) duration (0.5, 1.5, or 2.5 s), after which three green LEDs were switched on exactly on the same side where the mouse had made that first np. Afterward, the door opened for 5 s, allowing the mouse to drink as a response to a np. Any nosepoke during the delay period was considered as a premature or anticipatory response. During the training, premature responses had no consequence. However, during the testing phase, premature responses stopped the trial, requiring the mouse to leave the corner and start again. Therefore, animals had to learn to wait until the LED was on before making a second np.

Impulsivity was measured by the proportion of premature and correct responses (visits without premature responses).

#### Delay Discounting Task (DDT)

The delay discounting task (DDT) evaluates cognitive impulsivity. During this task, one of the bottles at each corner was filled with 2% sucrose water, while the other contained only water. In addition, while the doors leading to the water bottle could be opened immediately upon a np, there was a delay between the np and the opening of the sucrose doors; this delay started at 0.5 s and increased by 0.5 s every 24 h, reaching 8 s of delay at the end. Additional np at the closed sucrose door had no effect. Preference for sucrose and water bottles was measured. Increasing delays are expected to reduce the preference for sucrose; however, an earlier breakdown of sucrose preference in one of the groups would be taken as evidence for increased cognitive impulsivity.

#### Behavioral Data Analysis

The FlowR software (XBehavior, Bänk, Switzerland) was used to analyze the raw data obtained directly from the IntelliCage System software. To analyze the data from the adaptation phase, a saturated linear model was implemented to ANOVA, followed by linear model multiple comparisons as previously described in Fischer et al. (2017). With regard to the data from the place learning phase, RTT and DDT, the proportions of correct responses and premature responses were compared via generalized linear mixed models using a binomial link function on the logits, as described in Fournier et al. (2012) and Fischer et al. (2017). *F*-tests were taken for significance testing to take individual variation into account for error variance estimation, as proportions tended to be heavily overdispersed (Krackow et al., 2010; Fischer et al., 2017). For designs measuring individuals repeatedly, subject identity was added as random effect to the within-subject and between-subject fixed effects (giving essentially repeated measures ANOVA). Additionally, the raw data obtained by FlowR software were also used to analyze and plot the proportion of visits with nosepokes and proportion of visits with licks in the adaptation phase, using GraphPad Prism version 6.0. The normality of the data sets was verified using the Shapiro–Wilk normality test. Once a normal distribution was confirmed, genotype effects were analyzed by a two-tailed unpaired *t*-test; the level of significance was set to 0.05 (two-tailed) in all the statistical analyses. GraphPad Prism was also used to plot the sucrose preference in a timeline (delay-dependent) manner.

### Brain Tissue Preparation for Molecular Analysis

A cohort of *St3gal3* littermate HET (19 females, 7 males) and WT (13 females, 8 males) adult mice that were behaviorally naïve (age between 3 and 5 months old) were anesthetized with a lethal dose of isoflurane and sacrificed by cervical dislocation. Brains were dissected, frozen immediately in dry ice-cold isopentane and subsequently stored at −80°C until dissection of the areas of interest. Afterward, the left and right parts of the prefrontal cortex, hippocampus and striatum were separated on a pre-cooled plate, collected in separate vials and stored at −80°C for later use (see below).

### RNA Isolation and Quantitative RT-PCR

This analysis was done in the male and female sample sets. RNA was isolated from the left hemispheres of each brain region with the miRNeasy Mini Kit (Qiagen, Hilden, Germany). RNA integrity was verified by agarose gel electrophoresis. Complementary DNA (cDNA) was synthesized using the QuantiTect Reverse Transcription Kit (Qiagen, Hilden, Germany) using 600ng total RNA per reaction. We next proceeded with the qRT-PCR analysis to check the expression of the target genes *Cadm1*, *Cnp*, *Ncam1*, *St3gal3*, *St8Sia2*, *St8Sia4*, *Mag*, *Mog*, *Mbp*, *Cspg4*, *Olig2*, *Sox10*, *Plp1*, and *Plp2* (primer sequences for target genes can be consulted in Supplementary Table 1). *Rplp0*, *Ubc*, *B2m*, *Gapdh*, and *Actb2* served as reference genes for normalization of the gene expression. Reactions were run as previously described (Forero et al., 2020). PCR efficiencies for each primer pair were determined with LinRegPCR (Ruijter et al., 2009), using fluorescence raw data extracted from the CFX software. qbase + qPCR analysis software (Biogazelle, Zwijnaarde, Belgium) was used for calculation of relative expression values of all the target genes, using the threshold cycle values (Cq) from Bio-Rad CFX Maestro software (Bio-Rad Laboratories, Feldkirchen, Germany) and the primer efficiency values from LinReqPCR (Academic Medical Center, Amsterdam, Netherlands). These values were normalized by the most stable reference genes (Gapdh, *Actb* and *Rplp0* in hippocampus; *Ubc*, *B2m*, and *Rplp0* in PFC and striatum).

### Western Blot

Prefrontal cortex, hippocampal and striatal regions of the right hemispheres of the male sample set (WT = 8; HET = 7) were homogenized by sonication in Radio-Immunoprecipitation Assay (RIPA) buffer (Sigma-Aldrich, Darmstadt, Germany), supplemented with protease and phosphatase inhibitor tablets (Roche, Basel, Switzerland). A total of 20 μg of protein were loaded on 4–12% Bis-Tris protein gels (Thermo Fisher Scientific, Dreieich, Germany), electroblotted to a 0.45 μm pore nitrocellulose membrane (Thermo Fisher Scientific, Dreieich, Germany) and probed using one of the following primary antibodies, always in combination with either rabbit anti-α-tubulin antibody or goat anti- beta-tubulin (Abcam, Cambridge, United Kingdom) as loading controls: goat polyclonal anti-NCAM1/CD56 antibody (1:2,000, R&D Systems, Minneapolis, MA, United States), mouse monoclonal anti-PSA antibody (1:500, #NBP2-52710, Novus, Lingen, Germany), rabbit polyclonal anti-SynCAM1/2/3 (1:2000, Synaptic Systems, Göttingen, Germany), rabbit polyclonal anti-Myelin PLP (1:5,000, #ab28486, Abcam, Cambridge, United Kingdom), rabbit polyclonal anti-MBP (1:1,000, #PD004, MBL, Woburn, MA, United Kingdom). Finally, membranes were incubated with appropriate secondary fluorescent antibodies (all at 1:10,000; Li-Cor Biotechnology, Bad Homburg, Germany). Fluorescent band detection was conducted using the Fusion FX imaging system (Vilber Lourmat, Eberhardzell, Germany) and quantified using the Image Studio Lite software (Li-Cor Biotechnology, Bad Homburg, Germany). Target protein levels were normalized by the internal loading control and referred to average WT levels.

### Molecular Data Analysis

All statistical analyses as well as data graphs were performed using Prism version 9.2.0 (GraphPad Software, San Diego, CA, United States). Normal distribution was checked using the Shapiro–Wilk normality test and data were analyzed by two-way ANOVA or Student t-test with a level of significance of 0.05 (two-tailed).

## Results

### Heterozygous *St3gal3*-Deficient Mice Display Intermediate Levels of *St3gal3* mRNA

**FIGURE 2:**
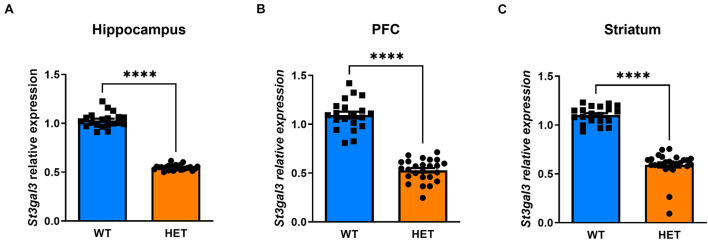
Relative expression of *St3gal3* in hippocampus, PFC and striatum of HET and WT mice. **(A)** Relative levels of *St3gal3* mRNA in the hippocampus (HET: *n* = 21, WT: *n* = 13). **(B)** Levels of *St3gal3* mRNA expression in the PFC (HET: *n* = 19, WT: *n* = 14). **(C)** Levels of *St3gal3* mRNA expression in the striatum (HET: *n* = 20, WT: *n* = 14). In all analyzed brain regions, a strongly significant decrease of around 50% in *St3gal3* expression was observed in heterozygote animals compared to their wildtype littermates (hippocampus: *t* = 29.59, *p* < 0.0001; PFC: *t* = 13.97, *p* < 0.0001; striatum: *t* = 14.62, *p* < 0.0001). Data were analyzed by Student’s *t*-test and presented as mean ± SEM. ^*⁣*⁣**^p < 0.0001. WT, wildtype; HET, heterozygote.

We first determined the expression of *St3gal3* in several brain regions of HET mice compared to WT littermates. By using PCR primers located in exons 2 (forward) and 3 (reverse) targeting specifically the *St3gal3* WT allele, we observed a gene dose dependent reduction of ∼50% in *St3gal3* levels in HET mice when compared to WT littermates, in all analyzed brain regions (*p* < 0.0001) (Figure 2).

### Heterozygous *St3gal3*-Deficient Mice Present With Sex-Dependent Alterations in Cognitive and Locomotor Activity

**FIGURE 3:**
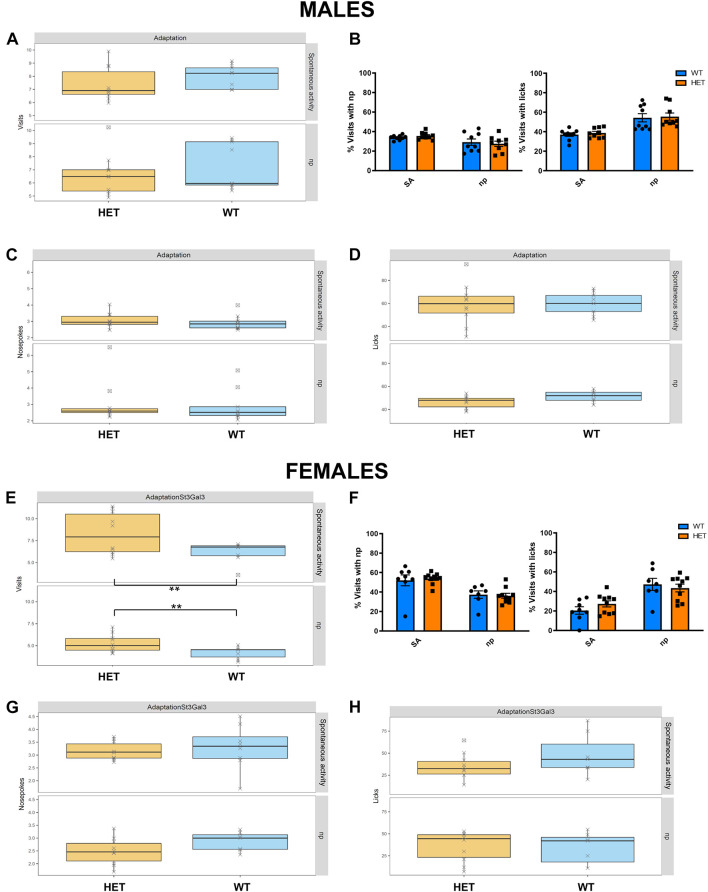
Assessment of exploratory behavior and association between nose-poking and water reward in HET mice. Adaptation phase was divided in spontaneous activity (SA) and nose-poke (Np) subphases, where the following parameters were assessed in the male cohort (*n* = 9/genotype) and the female cohort (*n* = 8 WT and 10 HET): **(A,E)** Number of visits/h; **(B,F)** proportion of visits with nosepokes and visits with licks; **(C,G)** mean number of nosepokes during visits with nosepokes and without licks; **(D,H)** median number of licks per visit. No genotype effects were observed in the male dataset. By contrast, female HET animals made significantly more visits compared to their WT littermates (*F*_1_,_31_ = 9.35, *p* = 0.0045) Data in panels **(B,F)** were analyzed by two-way ANOVA. Remaining data were analyzed and plotted with FlowR software. All data are presented as mean ± SEM.

We evaluated the effects of *St3gal3* haploinsufficiency using the Intellicage System, which allows the assessment of different behavioral domains that are related to ADHD pathophysiology. Profound differences between the male and the female *St3gal3*-deficient mice were observed. In HET males no significant genotype effects during the adaptation phase were detected. As shown in Figures 3A–D, the genotype had no effects on the number of visits, percentage of visits with either np or lick, number of np or number of licks, neither in the free adaptation subphase nor in the np subphase, suggesting unaltered activity and exploratory behavior in HET male mice. In contrast, female HET mice (Figures 3E–H) made significantly more visits per hour compared to their WT littermates during the entire adaptation phase (*p* = 0.0046), a finding that indicates enhanced exploratory behavior. However, the percentage of visits either with np or with np + licks remained unaltered.

**FIGURE 4:**
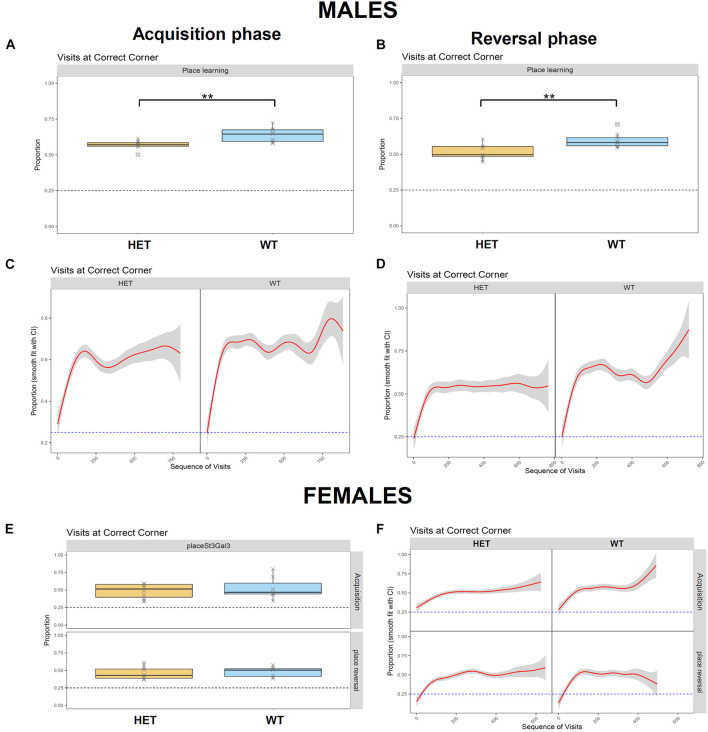
Assessment of learning performance and cognitive flexibility in HET mice. Place learning module was divided in two phases, Acquisition and Reversal, where the percentage of correct responses (visits to the correct corner) was used to evaluate learning performance of the male (*n* = 9/genotype) and the female (*n* = 8 WT and 10 HET) cohorts. **(A,B)** HET male mice displayed a reduced proportion of correct responses compared their WT littermates in Acquisition **(A)** (*F*_1_,_15_ = 12.35, *p* = 0,003128) and Reversal **(B)** (*F*_1_,_15_ = 11.97, *p* = 0,003496). **(C,D)** Cumulative visits at the correct corner in Acquisition **(C)** and Reversal **(D)**. **(E)** Averaged proportion of correct responses by the female cohort in Acquisition and Reversal phases, where no genotype effects were observed. **(F)** Cumulative visits at the correct corner by the female cohort in Acquisition and Reversal. Data were analyzed and plotted with FlowR software. Data in panels **(A,B,E)** presented as mean ± SEM. Dotted line on each graph indicates random expectation.

While ADHD is frequently associated with reduced cognitive performance, including impaired working memory that results in learning disabilities (Andersen et al., 2013; Tistarelli et al., 2020), *ST3GAL3* was also implicated in by a GWAS meta-analysis on general cognitive function (Davies et al., 2018). This deficit is also observed in null mutant mice (Yoo et al., 2015) and patients with loss-of-function mutations in *ST3GAL3* (Hu et al., 2011; Edvardson et al., 2013; Indellicato et al., 2020). These fact led us to evaluate learning and memory as well as cognitive flexibility in *St3gal3*-deficient mice in the place learning task. This module was divided into two phases, acquisition and reversal, in which the percentage of correct responses (visits to correct corner) was used to evaluate learning performance of the male and the female cohorts. Although all groups were able to learn the task and reached the learning criterion (set at 35% of correct responses, that is, random expectation plus 10%), HET male mice displayed a reduced averaged proportion of correct responses compared to their WT littermates in acquisition (Figures 4A,C; *p* = 0.0031) and reversal (Figures 4B,D; *p* = 0.0035); this effect was not observed in the female group, however (Figures 4E,F).

**FIGURE 5:**
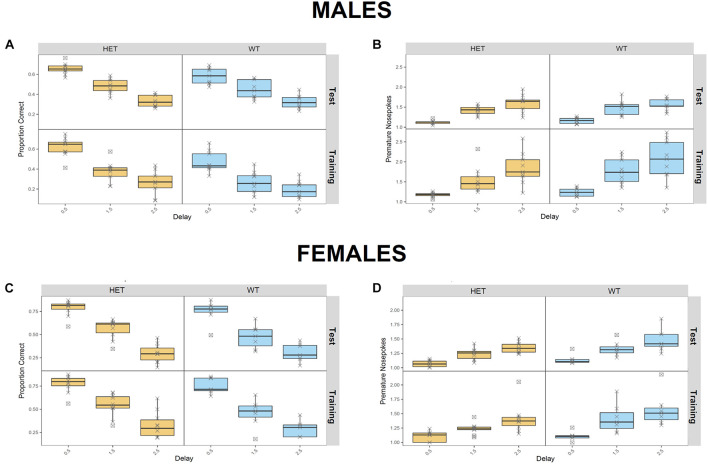
Assessment of motor impulvisity in *St3gal3* HET mice with the reaction time task. **(A,C)** Mean proportion of correct responses (depending on the delay between first and second nosepoke) made by males **(A)** and females **(C)** in the training and test phases. **(B,D)** Average number of premature nosepokes made by males **(B)** and females **(D)** in the training and test phases, depending on the delay between first and second nosepoke. A strongly significant effect of the delay on premature responses and correct responses was observed in both cohorts (*p* < 1e-08). In males, a genotype effect in the proportion of correct responses **(A)** was also detected in the test (*F*_1_,_47_ = 5.16, *p* = 0.02774) and training (*F*_1_,_47_ = 9.475, *p* = 0.003471) phases, but no effects in the number of premature responses were seen. In females, we observed a significant effect of the genotype x delay interaction in the proportion of correct responses (*F*_2_,_44_ = 4.624, *p* = 0.01504), as well as a significant genotype effect in the number of premature responses during the test phase (*F*_1_,_15_ = 5.236, *p* = 0.03706). Data were analyzed with FlowR using a generalised linear binomial modelling with random error estimation (*F*-test) to prevent significance overestimation due to overdispersion. Data are shown as mean ± SEM.

In the RTT, which evaluates motor impulsivity (Figure 5), a strongly significant effect of the delay on premature and correct responses was observed in both cohorts (*p* < 1e-08), as expected, with increased premature responses and reduced correct responses when the delay was higher. In addition, a genotype effect was observed in the male cohort in the proportion of correct responses (Figure 5A) in the test (*p* = 0.0277) as well as the training (*p* = 0.0035) phases. However, no effects in the number of premature responses were seen. On the other hand, in females, we observed a significant genotype x delay interaction in the proportion of correct responses (Figure 5C; *p* = 0.0150), with HET females having an increased number of correct responses only in shorter delays. Finally, significant genotype effect in the number of premature responses during the test phase was also detected in the female cohort (Figure 5D; *p* = 0.0370); in this case HET females made less premature responses in the test phase compared to their WT littermates; this finding was delay-independent and suggests an increased inhibitory control in the HET females.

The behavioral testing ended with the DDT, which assesses cognitive impulsivity (Supplementary Figure 2), however, no genotype effects were found.

### Heterozygous *St3gal3*-Deficient Mice Display Dysregulation of Genes Involved in Oligodendrogenesis, Myelin Formation and (Poly)sialylation

**FIGURE 6:**
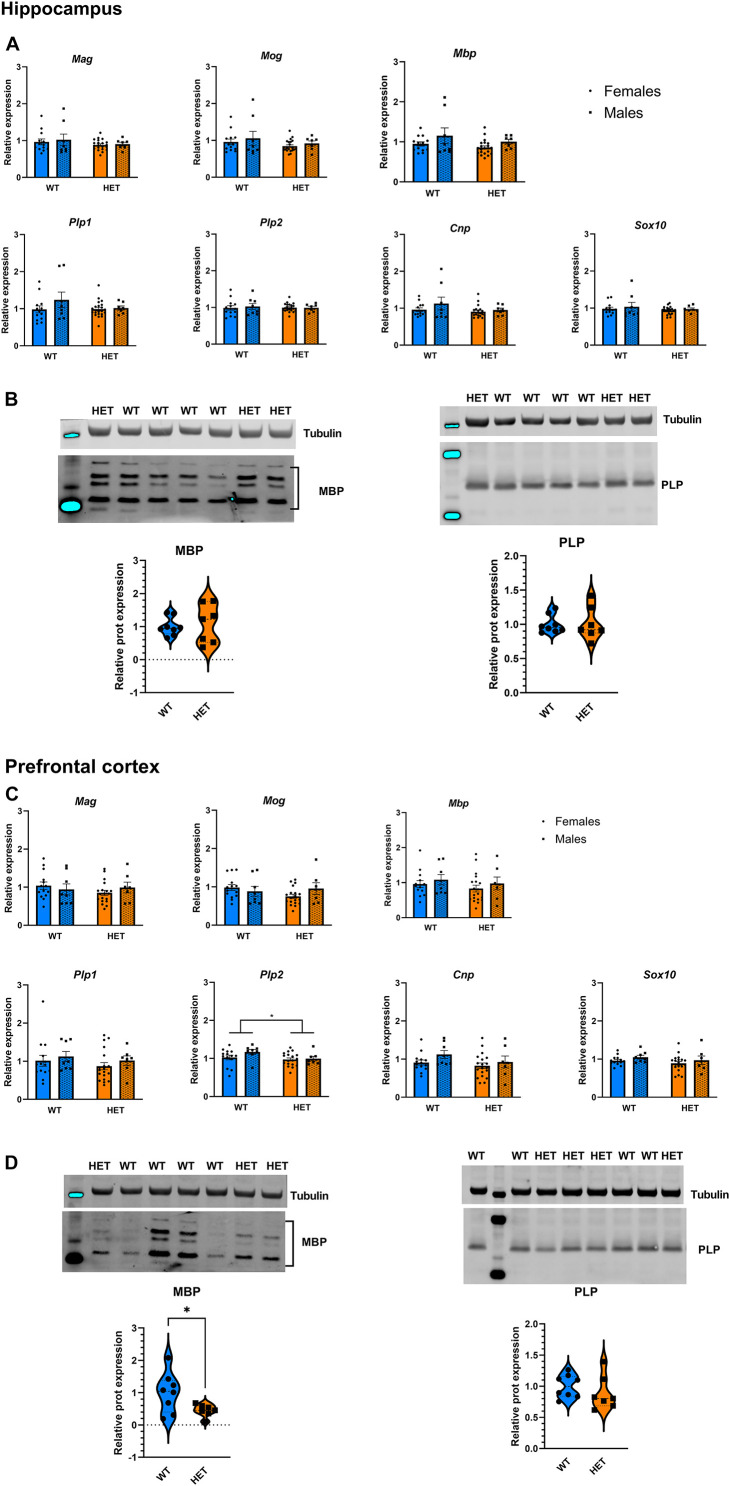
Expression analysis of genes involved in the myelination process. **(A)** Relative mRNA levels of 7 myelination biomarkers in the hippocampus of *St3gal3* mice (HET: *n* = 21, WT: *n* = 13). A trend toward a reduction of *Mbp* expression levels in females was observed (sex: *F*_1_,_43_ = 3.671, *p* = 0.062). **(B)** Protein levels of the myelination markers MBP and PLP in the hippocampus of *St3gal3* male mice (HET: *n* = 7, WT: *n* = 8). Representative blots are shown in each case. No differences were observed for any of the analyzed proteins. **(C)** Relative mRNA levels of 7 myelin biomarkers in the PFC of *St3gal3* mice (HET: *n* = 19, WT: *n* = 14). A significant genotype effect on *Plp2* levels was found (genotype: *F*_1_,_42_ = 5.142, *p* = 0.0286). A trend toward a reduction of *Plp1* expression levels in females (regardless from genotype) was also observed (sex: *F*_1_,_43_ = 3.936, *p* = 0.0537). **(D)** Protein levels of the myelination markers MBP and PLP in the PFC of *St3gal3* male mice (HET: *n* = 7, WT: *n* = 8). Representative blots are shown in each case. A significant reduction in MBP protein levels was observed in HET mice (*t* = 2.411, *p* = 0.0409). qPCR data were analyzed by two-way ANOVA and presented as mean ± SEM. Western blot data were analyzed by Student’s *t*-test with Welch’s correction and presented as violin plots with median + quartiles. *p < 0.05. For simplicity, only genotype effects are indicated in the figures.

**FIGURE 7:**
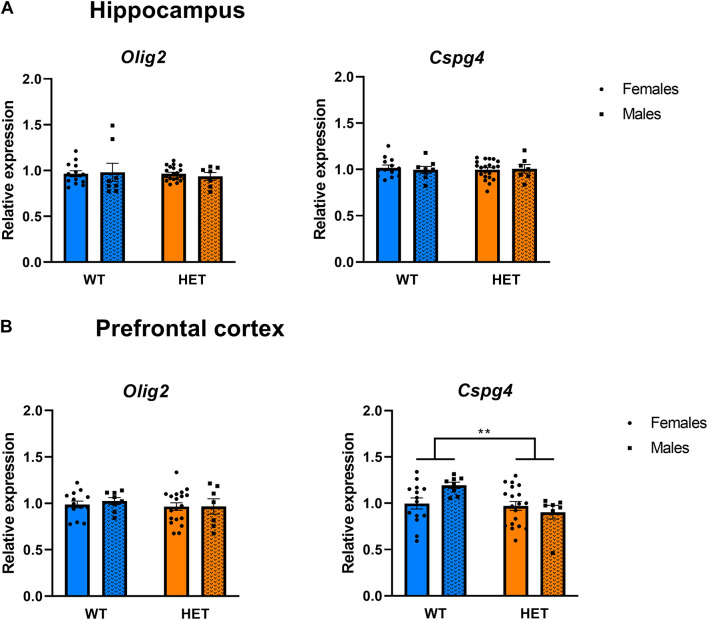
Expression analysis of genes involved in the generation and differentiation of oligodendrocytes. **(A)** Relative mRNA expression of the oligodendrocyte progenitor markers *Olig2* and *Cspg4* in the hippocampus of *St3gal3* mice (HET: *n* = 21, WT: *n* = 13). No significant differences were found. **(B)** Relative mRNA levelsexpression of the oligodendrocyte progenitor markers *Olig2* and *Cspg4* in the PFC (HET: *n* = 19, WT: *n* = 14). A significant effect of genotype on *Cspg4* expression levels was seen (genotype: *F*_1_,_42_ = 7.961, *p* = 0.0073). Data were analyzed by two-way ANOVA followed by Sidak *post hoc* test and presented as mean ± SEM. **p < 0.01.

We next assessed the effect of partial *St3gal3* deficiency on the expression of genes involved in oligodendrogenesis and myelin formation in three key brain regions involved in cognition and ADHD pathophysiology (PFC, hippocampus and striatum). We only found a genotype effect on *Plp2* levels in the PFC (*p* = 0.0286; Figure 6C), with HET animals having a reduced expression (average reduction of ∼10%) of this gene compared to WT mice. However, no genotype effects were observed in the hippocampus (Figure 6A) and striatum (Supplementary Figure 3). In addition, the expression of the oligodendrocyte marker *Cspg4/Ng2* was significantly reduced in the PFC of HET mice (*p* = 0.073; Figure 7B, average reduction in HET mice was 14%), an effect that was not observed in the hippocampus (Figure 7A). In both cases effects were mainly driven by the HET male dataset (16 and 25% reductions in the expression of *Plp2* and *Cspg4* in HET males compared to WT males, respectively). Trends for a sex effect on hippocampal *Mbp* (*p* = 0.062; Figure 6A) as well as PFC *Plp1* (*p* = 0.0537; Figure 6C) values were also detected.

Notably, Western blot analysis of MBP and myelin PLP as representative protein markers of myelination (Figures 6B,D) revealed no differences in PLP levels, but a significant decrease of ∼50% in PFC MBP levels in HET animals when compared to WT littermates (*p* = 0.0409; Figure 6D).

**FIGURE 8:**
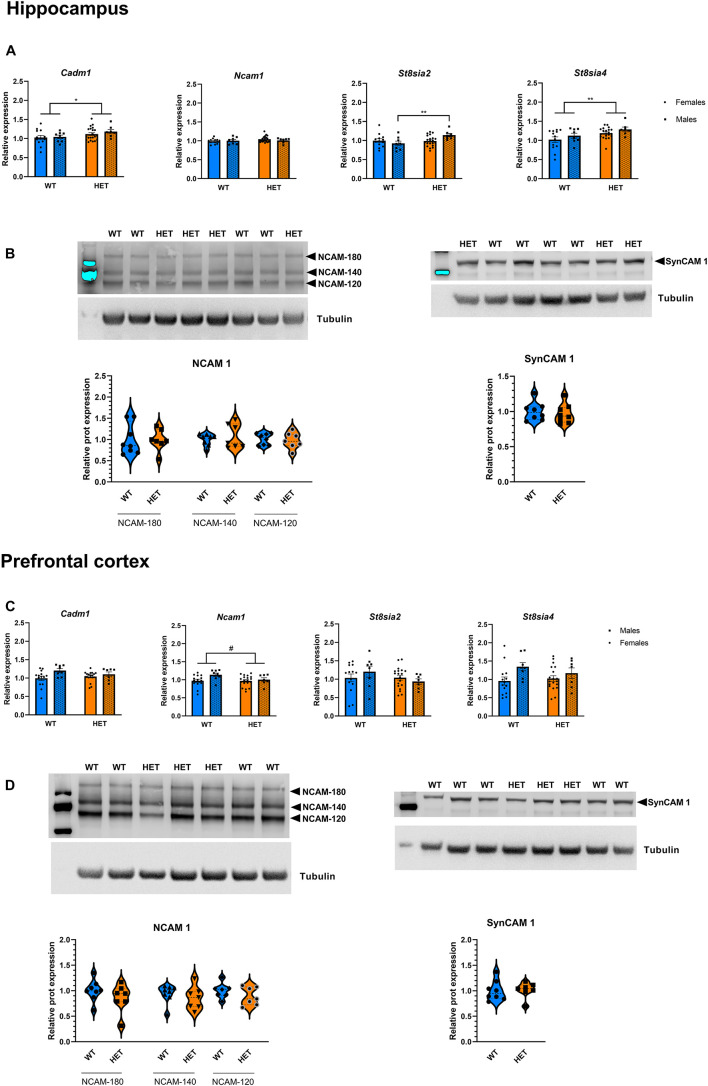
Expression analysis of genes involved in the polysialylation process. **(A)** Relative mRNA level of genes involved in the (poly)sialylation process in the hippocampus of *St3gal3* mice (HET: *n* = 21, WT: *n* = 13). A significant genotype effect on *Cadm1* levels was observed (genotype: *F*_1_,_43_ = 4.124, *p* = 0.0485). We detected a genotype main effect (genotype: *F*_1_,_43_ = 5.624, *p* = 0.0223) as well as a significant genotype × sex interaction (*F*_1_,_43_ = 4.736, *p* = 0.0351) on the levels of *St8sia2* expression; *post hoc* analysis revealed an upregulation in HET male mice compared to WT male mice (*post hoc p* = 0.0083) and HET females (*post hoc p* = 0.0481). A genotype effect was also observed for *St8sia4* (genotype: *F*_1_,_43_ = 7.538, *p* = 0.0088). **(B)** Protein levels of the adhesion molecules NCAM (isoforms NCAM-180, NCAM-140, and NCAM-120) and SynCAM 1 in the hippocampus of *St3gal3* male mice (HET: *n* = 7, WT: *n* = 8). Representative blots are shown in each case. No differences were observed for any of the analyzed proteins. **(C)** Relative mRNA level of genes involved in the polysialylation process in the PFC of *St3gal3* mice (HET: *n* = 19, WT: *n* = 14). A significant sex effect was observed in *Cadm1* (sex: *F*_1_,_42_ = 5.495, *p* = 0.0239). Trends toward sex and genotype effects were found for *Ncam1* (sex: *F*_1_,_42_ = 3.105, *p* = 0.0853; genotype: *F*_1_,_42_ = 2.981, *p* = 0.0916). A significant sex effect on *St8sia4* expression was also detected (*F*_1_,_42_ = 5.558, *p* = 0.0231). **(D)** Protein levels of the adhesion molecules NCAM (isoforms NCAM-180, NCAM-140, and NCAM-120) and SynCAM 1 in the hippocampus of *St3gal3* male mice (HET: *n* = 7, WT: *n* = 8). Representative blots are shown in each case. No differences were observed for any of the analyzed proteins. qPCR data were analyzed by two-way ANOVA followed by Sidak *post hoc* test (when necessary) and presented as mean ± SEM. Western blot data were analyzed by Student’s *t*-test with Welch’s correction and presented as violin plots with median + quartiles. ^#^*p* < 0.1, *p < 0.05, **p < 0.01. For simplicity, only genotype effects are indicated in the figures.

In addition, *St3gal3* deficiency was associated with expression changes of several genes involved in the process of (poly)sialylation. In the hippocampus (Figure 8A), we detected a significant genotype effect on *Cadm1* levels, with HET animals displaying higher levels (∼10% increase) compared to their WT littermates (*p* = 0.0485). Moreover, ANOVA detected a genotype main effect (*F*_1_,_43_ = 5.624, *p* = 0.0223) as well as sex × genotype interaction (s × g: *F*_1_,_43_ = 4.736, *p* = 0.0351) that affected hippocampal *St8sia2* expression. While *St8sia2* levels were generally higher in HET animals compared to WT, *post hoc* analysis revealed that *St8sia2* expression was particularly upregulated in HET males when compared to HET females (∼14% increase, *p* = 0.0481) and WT males (∼23% increase, *p* = 0.0083). Finally, the hippocampal expression of the other polysialyltransferase included in the study, *St8sia4*, was also significantly increased in HET mice compared to their WT littermates (∼16% increase, *p* = 0.0088).

In the PFC (Figure 8C), effects were generally modest, only sex main effects in the expression of *Cadm1* (*p* = 0.0239) and *St8sia4* (*p* = 0.0231) were observed. Trends toward significant effects of genotype and sex on *Ncam1* expression were also seen (sex: *p* = 0.0853; genotype: *p* = 0.0916). As for striatum, a trend toward a sex effect was observed for *St8sia4* (Supplementary Figure 2; *p* = 0.0657).

**FIGURE 9:**
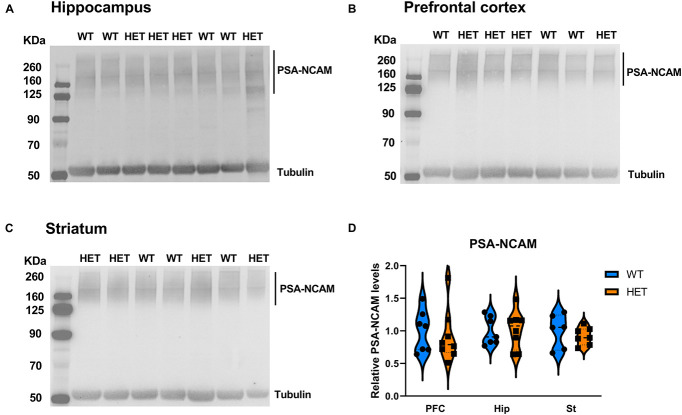
Analysis of polysialylated proteins in different brain regions of the mouse brain. **(A–C)** Representative images of immunoblots of PSA in hippocampus **(A)**, PFC **(B)**, and Striatum **(C)**. **(D)** Quantification of PSA levels, normalized to α-tubulin (HET: *n* = 8, WT: *n* = 7). Data were analyzed by two-way ANOVA and presented as mean ± SEM.

The evidence that HET animals display an upregulation of genes involved or affected by the process of polysialylation led to us to investigate whether the levels of the main targets of polysialylation (NCAM1, SynCAM1) were altered in those mice (Figure 8). We also analyzed global PSA levels in polysialylated proteins, but Western blot analyses revealed no differences, neither in the levels of total NCAM1, total SynCAM1 nor in PSA-glycoproteins in the PFC, hippocampus and striatum of HET mice, compared to their WT littermates (Figure 9).

## Discussion

Our results demonstrate how partial reduction in *St3gal3* expression produces critical changes at the molecular and behavioral level, with a specific involvement of genes central to the myelination process and the formation of PSA-glycoproteins.

Despite representing the most common NDD, the search for genetic risk factors for ADHD has been a considerable challenge. Therefore, the identification of risk variants surpassing genome-wide significance has provided new sources of hypothesis-generating research (Demontis et al., 2019). Among several other ADHD risk genes, *ST3GAL3*, previously related to several severe intellectual disability (ID)-presenting conditions (Hu et al., 2011; Edvardson et al., 2013; Indellicato et al., 2020), encodes an enzyme that is crucial for sialoglycan formation and therefore contributes to critical processes in the mammalian forebrain, such as sialylation of glycoproteins, oligodendrogenesis and ultimately myelin formation, with numerous roles in cell adhesion, synaptic plasticity and neuronal communication (Kolter et al., 2002; Wang, 2009; Audry et al., 2011; Yoo et al., 2015).

In concordance with the human phenotypes caused by loss-of-function mutations, *St3gal3* null mutant mice are characterized by marked cognitive deficits, reduced motor coordination and hyperactive behavior (Yoo et al., 2015) as well as spontaneous non-lethal epileptic seizures (Jamey Marth, personal communication; also see: www.jax.org/strain/006899). All these profound abnormalities might be the consequence of a disrupted myelination process. The question remains how severe neurological and cognitive alterations resulting from ST3GAL3 homozygous inactivation help to understand ADHD-related pathophysiology? Indeed, deficient ST3GAL3 activity may be a key factor in ADHD pathogenesis for several reasons. First, dysregulated myelin formation may compromise the developmental trajectory leading to ADHD (Lesch, 2019). Several diffusion tensor imaging (DTI) studies and meta-analyses revealed alterations in white matter integrity assessed by fractional anisotropy in cortico-striatal tracts and commissural tracts of the corpus callosum, all of them relevant for ADHD pathophysiology as well as to ADHD-associated impairment of cognitive performance (van Ewijk et al., 2012; Chen et al., 2016). Thus, our findings support the notion that a more subtle alteration in ST3GAL3 function, e.g., through common risk variants, may underlie distinct dimensions of ADHD symptomatology, such as inattention or working memory impairment, whereas loss-of-function mutations lead to severe phenotypes. In agreement with this notion, a recent study found that a large set of ID-causing genes, including *ST3GAL3*, contributes to ADHD risk via common variants (Klein et al., 2020).

Experimental animal models represent an invaluable source of information to explore the contribution of individual genes to human disorders (Homberg et al., 2021). However, null mutant models exhibiting a complete inactivation of a given gene may be inadequately informative in the case of genetically complex diseases, in which each risk gene contributes to vulnerability only modestly. We consequently focused our study on *St3gal3* HET mice. Our results show that HET mice display a gene dose-dependent 50% reduction in *St3gal3* expression. Interestingly, such a reduction was sufficient to give rise to expression changes of several genes and a myelination-related protein. In the PFC, the effects of *St3gal3* deficiency were largely restricted to myelination markers, with downregulation of *Plp2* and *Cspg4* as well as a 50% reduction in MBP, a major constituent of the myelin sheath in brain. Consistent with our observation, a previous study revealed that the expression level of MBP, was also reduced in the brain of *St3gal3* null mutant mice, along with a significant reduction in the number of myelinated axons in the corpus callosum (Yoo et al., 2015). The fact that partial loss of St3gal3 resembles the molecular signatures of complete depletion further highlights the critical role of ST3GAL3 function in brain plasticity.

In the hippocampus of *St3gal3*-deficient mice, the strongest expression differences were observed for several genes related to glycoprotein sialylation. Specifically, the two polysialyltransferases, *St8sia4* and *St8sia2* (responsible for the attachment of polySia to several adhesion molecules) were upregulated. Interestingly, several association studies have identified *ST8SIA2* (also known as *SIAT8B*) as a risk gene for several neurodevelopmental and psychiatric disorders, including autism spectrum disorder (ASD) (Anney et al., 2010), bipolar disorder (McAuley et al., 2012) and schizophrenia (Arai et al., 2006; Tao et al., 2007; McAuley et al., 2012). These findings are further supported by the identification of loss-of-function mutations implicating *ST8SIA2* in patients with schizophrenia (Isomura et al., 2011) and ASD with epilepsy (Kamien et al., 2014). A *ST8SIA2* haplotype has also been shown to have differential effects in patients with psychotic disorders and healthy subjects on white matter integrity of the parietal lobe (Fullerton et al., 2018). In addition, *St8sia2* null mutant mice display behavioral symptoms that resemble those from patients with psychoses, such as cognitive dysfunction, deficits in prepulse inhibition, and increased sensitivity to amphetamine-induced locomotion (Kröcher et al., 2015). The upregulation of *St8sia4* and *St8sia2* observed in the brain of *St3gal3* HET mice may be interpreted as a compensatory mechanism to the reduction in *St3gal3* expression, in order to maintain the levels of PSA glycoproteins such as PSA-NCAM1 or PSA-SynCAM1 (PSA-CADM1). Indeed, in our study *Cadm1* expression was also upregulated in the hippocampal region of *St3gal3* HET mice, possibly as a result of the same compensatory mechanism. CADM1 is essential for many neurodevelopmental processes including myelination (Niederkofler et al., 2010). Additionally, a study with a mouse model harboring a dominant negative mutation in *Cadm1* underscores a relationship between CADM1 function and ADHD-like behaviors (Sandau et al., 2012). Of note, CADM1 shows broadly overlapping expression with NCAM1 during brain development, but its protein product SynCAM1 is specifically polysialylated in the NG2 cells (Schnaar et al., 2014), which are considered progenitor cells types for oligodendrocytes, astrocytes, and neurons (Dimou and Gallo, 2015). Probably in cojunction with the upregulation of *Cadm1* and the polysialyltransferases *St8sia4* and *St8sia2*, *St3gal3* HET mice present with a downregulation of *Cspg4*, which encodes the proteoglican NG2, the only common marker of all NG2-specific cells (Dimou and Gallo, 2015).

Nevertheless, despite the robust alterations in the mRNA expression of several genes related to polysialylation, we did not detect significant changes at the protein level, neither for PSA-glycoproteins nor for total levels of SynCAM1 or NCAM1. This is in contrast with the increase in PSA glycoproteins observed in *St3gal3* full knockout mice (Yoo et al., 2015). There are several explanations to our negative finding. On the one hand, the upregulation of *St8sia4* and *St8sia2* could counteract the effects of *St3gal3* partial deficiency. On the other hand, the fact that *St3gal3* deficiency has a greater impact on *Cadm1* expression rather than on *Ncam1* (which encodes the main PSA acceptor) could explain why total PSA levels are not modified in those mice.

The stronger effects on the sialylation-related genes in the hippocampus compared to PFC might be linked to the higher rates of adult neurogenesis described in the hippocampus in comparison to other brain regions. In agreement, PSA is restricted in adulthood to regions with ongoing neurogenesis and synaptic plasticity (Galuska et al., 2010; Schnaar et al., 2014).

Strikingly, *St3gal3*-deficient mice presented with various behavioral alterations resembling deficits observed in ADHD patients such as locomotor activity and cognitive performance. In this regard, male HET mice showed impaired memory in the place learning task and, it is well known that learning disabilities are frequently observed in ADHD patients (Andersen et al., 2013; Tistarelli et al., 2020) and also supported by a GWAS meta-analysis implicating *ST3GAL3* in general cognitive function (Davies et al., 2018). This deficit is also observed in null mutant mice (Yoo et al., 2015) and patients with loss-of-function mutations in *ST3GAL3* (Hu et al., 2011; Edvardson et al., 2013; Indellicato et al., 2020). On the other hand, HET females demonstrated increased locomotor activity. As hyperactivity is one of the core features of ADHD, together with inattention and increased impulsivity (Tistarelli et al., 2020), moderate deficiency in *St3gal3* expression in female HET mice is able to capture some of the most representative features of ADHD. Unexpectedly, HET mice appeared to display increased inhibitory control (increased percentage of correct responses in the RTT). In agreement with such an increased inhibitory control, we also observed that HET females made a reduced number of premature nosepokes per visit during the test phase of the RTT. Our behavioral findings suggest that the contribution of *ST3GAL3* to ADHD may be restricted to distinct behavioral domains, such as cognitive performance, learning abilities and locomotor activity, whereas other traits (attention, impulsivity) are affected to a lesser extent. However, we cannot rule out that the effects of *St3gal3* deficiency on impulsivity or attention might be different in younger animals or under stressful conditions that may act as a second-hit trigger for other dysfunctional behavioral phenotypes.

Another feature of our findings is the sexual dimorphism. The locomotor hyperactivity in *St3gal3* HET females is in contrast to the clinical observation of female patients with ADHD, which tend to show less hyperactivity and more inattention, when compared to males diagnosed with ADHD. In addition, males normally suffer more often from comorbid disorders implicating impaired cognitive control, such as oppositional defiant, conduct or drug use disorders (Mansouri et al., 2016). However, because male patients with ADHD tend to display more externalizing behaviors, ADHD among females remains underdiagnosed (Slobodin and Davidovitch, 2019). Together, these behavioral characteristics may be explained by sex-specific differences at the molecular level that impact brain (cyto)architecture and synaptic plasticity (Manoli and Tollkuhn, 2018). Accordingly, we identified several genes that were differentially expressed in males and females, such as *Cadm1*, *St8sia4* and *Ncam1* in the PFC, among others.

In conclusion, our findings indicate that haploinsufficiency of ST3GAL3 results in a sex-dependent alteration of cognition, behavior and markers of brain plasticity. The *St3gal3*-haplodeficient mouse proved to represent a remarkably informative model to better understand the contribution of *ST3GAL3* variation to ADHD risk. Our results thus support ST3GAL3 as a key hub in the pathway toward innovative strategies to prevent or treat cognitive and behavioral deficits related to ADHD pathophysiology.

## Data Availability Statement

The raw data supporting the conclusions of this article will be made available by the authors, without undue reservation.

## Ethics Statement

The animal study was reviewed and approved by Government of Lower Franconia.

## Author Contributions

OR, MF, and K-PL developed the conception and design of the study. OR, JA-R, PA, and H-PK performed the behavioral experiments. JA-R, GO, H-PK, DD, and DH performed the molecular experiments. JA-R, MF, OR, and H-PK performed the statistical analysis of behavioral data. JA-R and OR performed the statistical analysis of the molecular data. OR led the manuscript writing. OR and JA-R wrote the manuscript. All authors contributed to manuscript revision, critically read and approved the submitted version.

## Conflict of Interest

The authors declare that the research was conducted in the absence of any commercial or financial relationships that could be construed as a potential conflict of interest.

## Publisher’s Note

All claims expressed in this article are solely those of the authors and do not necessarily represent those of their affiliated organizations, or those of the publisher, the editors and the reviewers. Any product that may be evaluated in this article, or claim that may be made by its manufacturer, is not guaranteed or endorsed by the publisher.

## Footnotes

1
https://www.jax.org/
