# Supplementary material for: Haploinsufficiency of the Attention-Deficit/Hyperactivity Disorder Risk Gene St3gal3 in Mice Causes Alterations in Cognition and Expression of Genes Involved in Myelination and Sialylation
Source: Front Genet. 2021 Sep 28;12:688488. doi: 10.3389/fgene.2021.688488 (PMC8505805; doi:10.3389/fgene.2021.688488)
Supplement: Supplementary file 1 [file Data_Sheet_1.PDF]

Supplementary Material

Supplementary Table 1. Oligonucleotide sequences of the target genes used for qPCR analysis. All primer sequences were designed with Primer- Blast (Ye et al., 2012).

Gene name	Type	Oligonucleotide sequence (5'→3')	Function
Cspg4	oligodendrocyte progenitor marker	F: GACCAACCCCCTGTTCTCAC<br>R: TGGGCCCGAATCATTGTCTG	Encodes NG2 protein. Involved in oligodendrocyte generation, developmental myelination, as well as myelin repair (Nishiyama et al., 2002; Galuska et al., 2010)
Olig2	oligodendrocyte progenitor marker	F: GAAGCGATGGAGATGCG<br>R: CCCAGACCCTTGGAGTGTTC	Determines motor neuron and oligodendrocyte differentiation (Zhang et al., 2015)
Cnp	myelination marker	F: GCTTGAGCTGGTCAGCTACTT<br>R: TTGAAGGCCTTGCCATACGA	Encodes a major component of myelin: its deficiency causes white matter degeneration (Al-Abdi et al., 2020)
Mag	myelination marker	F: TTCTCAGGGGGAGACAACC<br>R: ACTCTCCTGGGGCTCTCAGT	It is involved in myelination during nerve regeneration in the PNS and is vital for long-term survival of myelinated axons following myelinogenesis (Quarles, 2007).
Mbp	myelination marker	F: CTCCCTGCCCCAGAAGTC<br>R: GAGGTGGTGTTCGAGGTGTC	Maintains the correct structure of myelin, interacting with the lipids in the myelin membrane (Boggs, 2006).
Mog	myelination marker	F: CTGGCAGGACAGTTTCTTGA<br>R: AAAGAGGCCAATGGGAAATC	It is important for myelination of nerves in the CNS. It influences white matter development (Wu et al., 2018).
Plp1	myelination marker	F: AGTCAGCCGCAAAACAGACTA<br>R: CCCCTACCAGACATCTAGCAC	May play a role in the compaction, stabilization and maintenance of myelin sheaths (Boiko and Winckler, 2006).
Plp2	myelination marker	F: ATGGCGGATTCTGAGCGTC<br>R: AGGCCGATGTTGTAGATGCAC	It conveys an increased risk to intellectual disability and may play a role alleviating cellular stress (Zhang et al., 2015).
Sox10	myelination marker	F: ATGTCAGATGGGAACCCAGA<br>R: CGGACTGCAGCTCTGTCTTT	Important for neural crest and peripheral nervous system (PNS) development (Weider et al., 2013).
Ncam1	Related to (poly)sialylation	F: AGGCCGAATACGTCTGCATC<br>R: CCTCTAGTTCCATGGCCGTC	Implicated in neurite outgrowth, cell-cell adhesion synaptic plasticity, learning and memory. In mammals, 95% of *Ncam1* is linked to PSA (Schnaar et al., 2014).
Cadm1	Related to (poly)sialylation	F: AACCGCAAGTGCATATCCAGA<br>R: CGGCATGTTGAGGCATTTCA	Encodes SynCAM1. Potent inducer of synapse formation. It shows a broadly overlapping expression with NCAM1 during brain development, but receives PSA only in a distinct cellular subset, NG2 glia cells (Galuska et al., 2010).
ST8Sia2	Related to (poly)sialylation	F: TGGCCAGGAGATTGACACAC<br>R: CCGGGCATACTCCTGAACTG	Transfers sialic acids to nascent oligosaccharides. Together with *ST8Sia4*, *ST8Sia2* links each sialic acid residue to each other generating PSA (Mori et al., 2017).
ST8Sia4	Related to (poly)sialylation	F: CTGGCTCCACCATCTTCCAAC<br>R: GCTCTTGACCACAGATACGTCA	Transfers sialic acids to nascent oligosaccharides. Together with *ST8Sia2*, *ST8Sia4* links each sialic acid residue to each other generating PSA. Acts together with *ST8Sia2* (Mori et al., 2017).
St3Gal3	Related to (poly)sialylation	F: GGGACTCTTGGTATTTGTGCG<br>R: CGGAGTCAAGGGAAAGAAGCA	Transfers sialic acids to nascent oligosaccharides. It attaches the first sialic acid residue, to which other 8-100 sialic acids residues can link in order to generate PSA (Chung et al., 2015).

Abbreviations: F: forward; R: reverse.

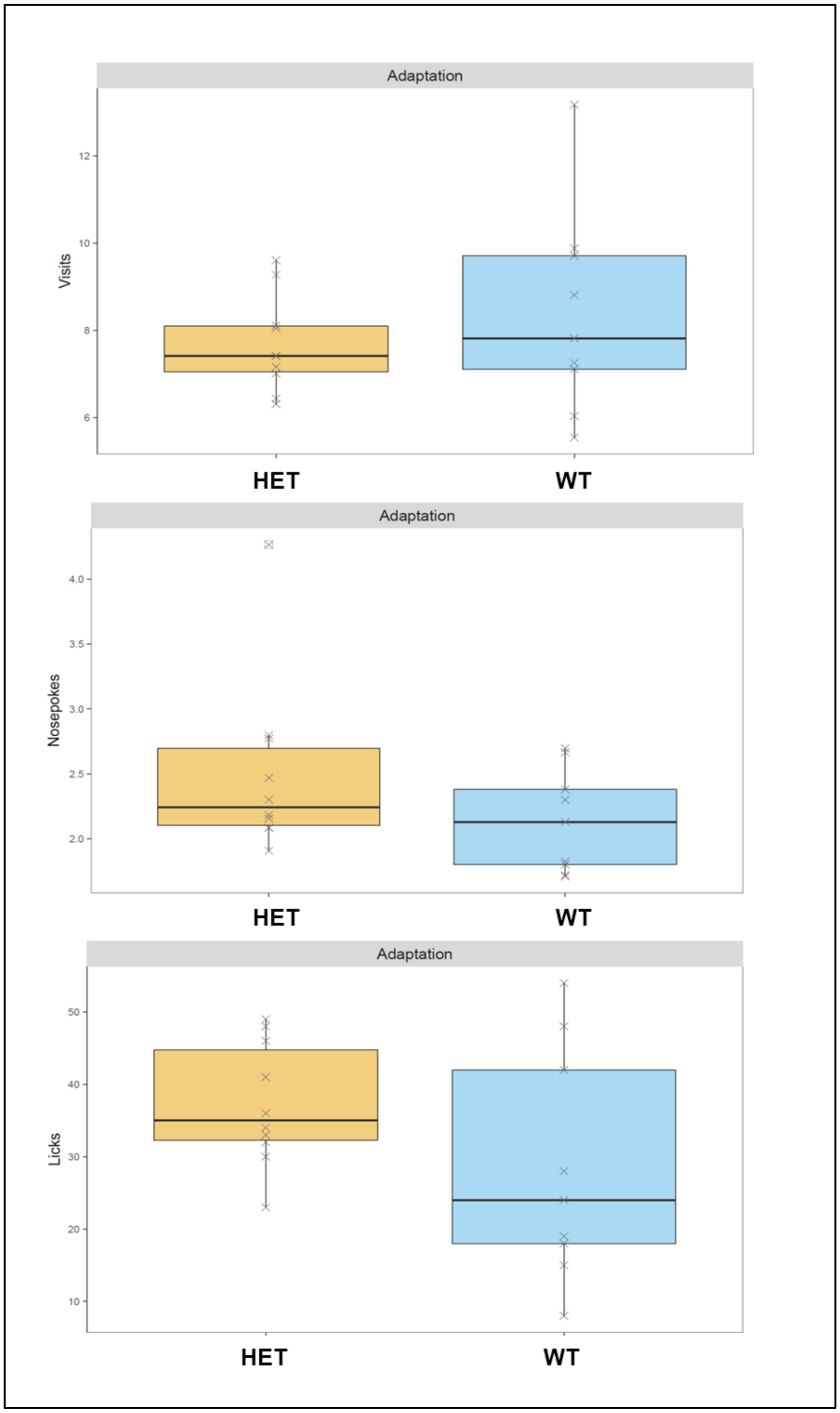

Supplementary figure 1. Measurement of exploratory behavior and association between nose-poking and water reward during the second adaptation phase. The male cohort (n = 9/genotype) was subjected to a second adaptation which was only composed by a nosepoke (Np) phase. The figure displays the number of visits/h, mean number of nosepokes during visits with nosepokes and without licks, as well as median number of licks per visit. No genotype effects were observed in the male cohort during this phase. Data were analyzed and plotted with FlowR software.

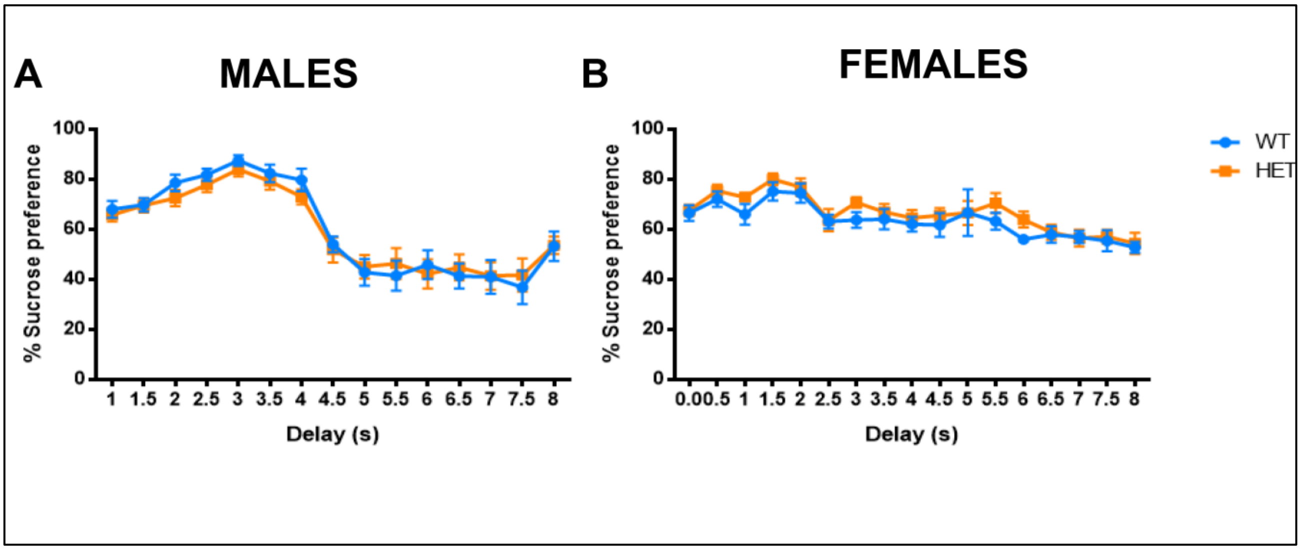

Supplementary figure 2. Delay discounting task: assessment of cognitive impulsivity in *St3gal3* HET mice. Percentage of sucrose preference in males (A; n = 9/genotype) and females (B; n = 7 WT and 10 HET) throughout the whole task. Sucrose delay increased 0.5 s/24 h from 0 to 8 s. There were no significant differences between HET mice and their WT littermates. (n = 9/genotype). Data were analyzed by two-way ANOVA with repeated measures and presented as mean ± SEM.

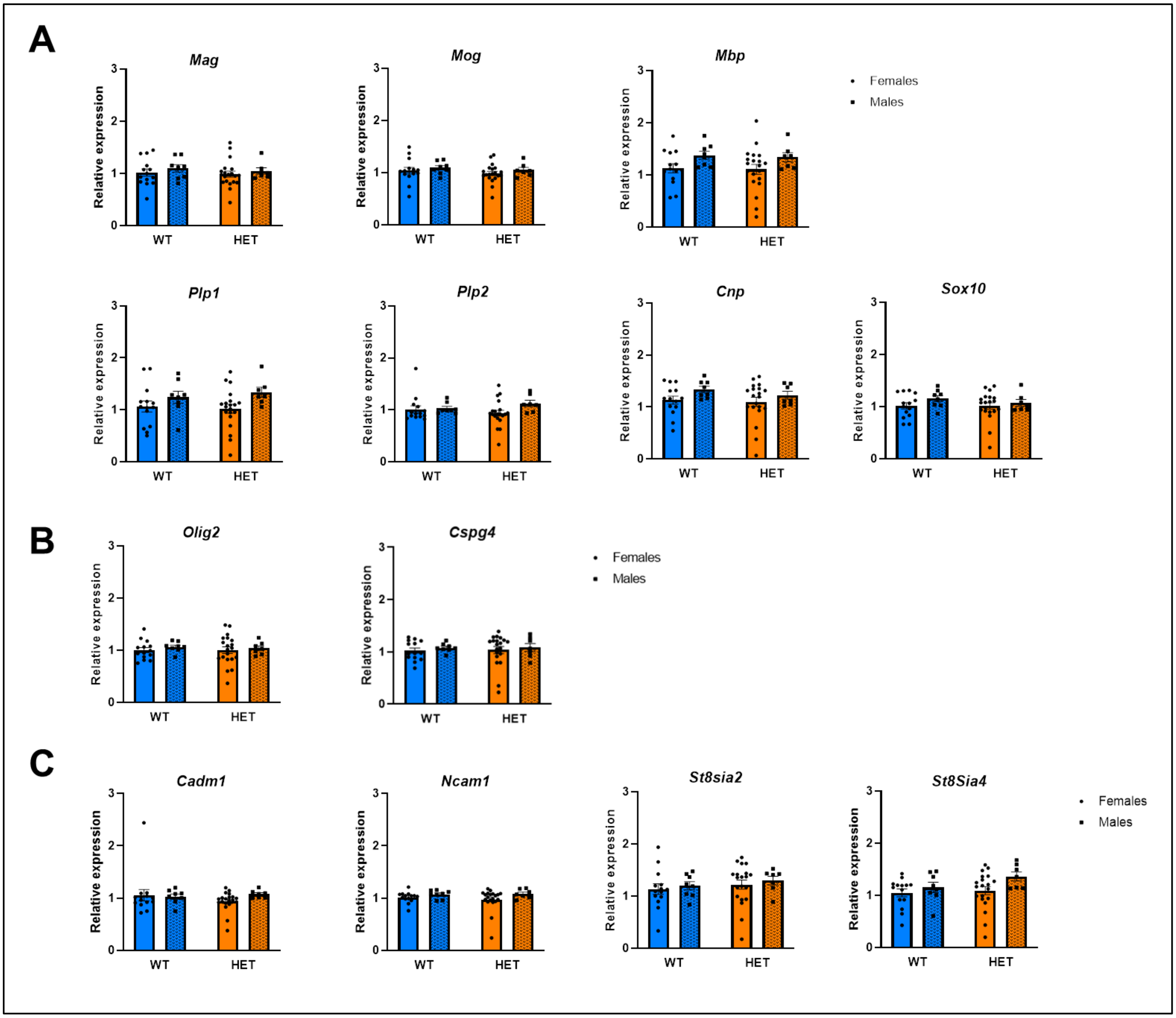

Supplementary figure 3. Expression analysis in the striatum of *St3gal3* mice (HET: n = 20, WT: n = 14)**. *(A)*** Relative expression of 7 myelination biomarkers. No significant differences were observed. (B) Relative expression levels of the oligodendrocyte progenitor markers *Olig2* and *Ng2*. No differences were observed. (C) Relative expression levels of genes involved in the polysialylation process. A trend towards a sex effect on *St8sia4* levels was observed ($F_{1,43} = 3.568$, $p = 0.0657$). Data were analyzed by two-way ANOVA followed by Sidak post-hoc test and presented as mean ± SEM. For simplicity, only genotype effects are indicated in the figures.

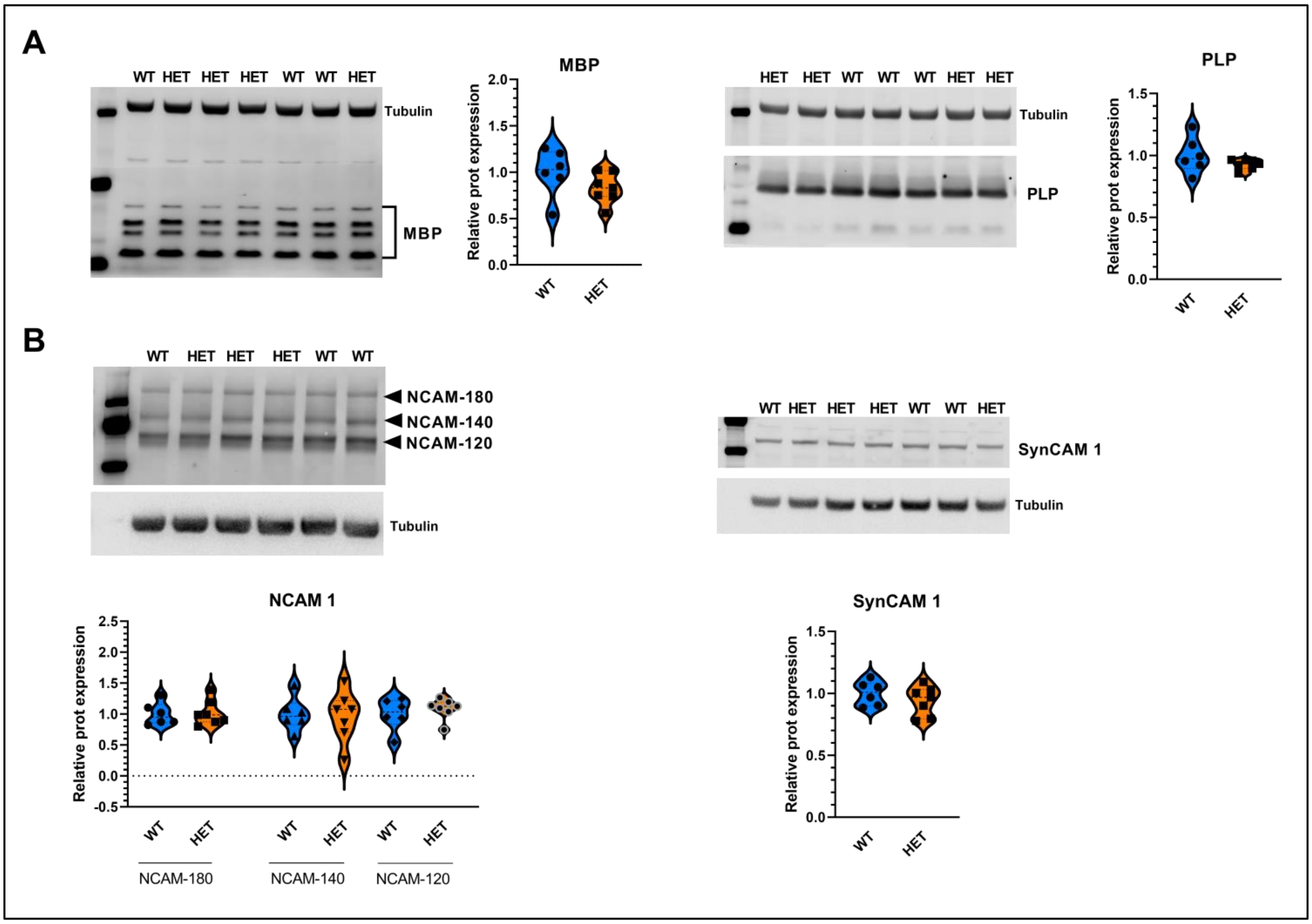

Supplementary figure 4. Protein levels of the myelination markers MBP and PLP (panel A) and the adhesion molecules NCAM (isoforms NCAM-180, NCAM-140 and NCAM-120) and SynCAM 1 (panel B) in the Striatum of *St3gal3* male mice (HET: n= 7, WT: n = 8). Representative blots are shown in each case. No differences were observed for any of the analyzed proteins. Data were analyzed by Student t test with Welch's correction and presented as violin plots with median + quartiles.

Supplementary references

Al-Abdi, L., Al Murshedi, F., Elmanzalawy, A., Al Habsi, A., Helaby, R., Ganesh, A., et al. (2020). CNP deficiency causes severe hypomyelinating leukodystrophy in humans. *Human genetics*, *139*(5), 615–622. doi: 10.1007/s00439-020-02144-4

Boggs J. M. (2006). Myelin basic protein: a multifunctional protein. *Cellular and molecular life sciences : CMLS*, *63*(17), 1945–1961. doi: 10.1007/s00018-006-6094-7

Boiko, T., and Winckler, B. (2006). Myelin under construction -- teamwork required. *The Journal of cell biology*, *172*(6), 799–801. doi: 10.1083/jcb.200602101

Chung, C. Y., Yin, B., Wang, Q., Chuang, K. Y., Chu, J. H., and Betenbaugh, M. J. (2015). Assessment of the coordinated role of ST3GAL3, ST3GAL4 and ST3GAL6 on the α2,3 sialylation linkage of mammalian glycoproteins. *Biochemical and biophysical research communications*, *463*(3), 211–215. doi: 10.1016/j.bbrc.2015.05.023

Galuska, S. P., Rollenhagen, M., Kaup, M., Eggers, K., Oltmann-Norden, I., Schiff, M., et al. (2010). Synaptic cell adhesion molecule SynCAM 1 is a target for polysialylation in postnatal mouse brain. *Proceedings of the National Academy of Sciences of the United States of America*, *107*(22), 10250–10255. doi: 10.1073/pnas.0912103107

Quarles R. H. (2007). Myelin-associated glycoprotein (MAG): past, present and beyond. *Journal of neurochemistry*, *100*(6), 1431–1448. doi: 10.1111/j.1471-4159.2006.04319.x

Mori, A., Hane, M., Niimi, Y., Kitajima, K., and Sato, C. (2017). Different properties of polysialic acids synthesized by the polysialyltransferases ST8SIA2 and ST8SIA4. *Glycobiology*, *27*(9), 834–846. doi: 10.1093/glycob/cwx057

Nishiyama, A., Watanabe, M., Yang, Z., and Bu, J. (2002). Identity, distribution, and development of polydendrocytes: NG2-expressing glial cells. *Journal of neurocytology*, *31*(6-7), 437–455. doi: 10.1023/a:1025783412651

Schnaar, R. L., Gerardy-Schahn, R., and Hildebrandt, H. (2014). Sialic acids in the brain: gangliosides and polysialic acid in nervous system development, stability, disease, and regeneration. *Physiological reviews*, *94*(2), 461–518. doi: 10.1152/physrev.00033.2013

Weider, M., Reiprich, S., and Wegner, M. (2013). Sox appeal - Sox10 attracts epigenetic and transcriptional regulators in myelinating glia. *Biological chemistry*, *394*(12), 1583–1593. doi: 10.1515/hsz-2013-0146

Wu, F., Kong, L., Zhu, Y., Zhou, Q., Jiang, X., Chang, M., et al. (2018). The Influence of Myelin Oligodendrocyte Glycoprotein on White Matter Abnormalities in Different Onset Age of Drug-Naïve Depression. *Frontiers in psychiatry*, *9*, 186. doi: 10.3389/fpsyt.2018.00186

Ye, J., Coulouris, G., Zaretskaya, I., Cutcutache, I., Rozen, S., and Madden, T. L. (2012). Primer-BLAST: a tool to design target-specific primers for polymerase chain reaction. *BMC bioinformatics*, *13*, 134. doi: 10.1186/1471-2105-13-134

Zhang, L., Wang, T., and Valle, D. (2015). Reduced PLP2 expression increases ER-stress-induced neuronal apoptosis and risk for adverse neurological outcomes after hypoxia ischemia injury. *Human molecular genetics*, *24*(25), 7221–7226. doi: 10.1093/hmg/ddv422
